# Increase of Hemoglobin Levels by Anti-IL-6 Receptor Antibody (Tocilizumab) in Rheumatoid Arthritis

**DOI:** 10.1371/journal.pone.0098202

**Published:** 2014-05-30

**Authors:** Motomu Hashimoto, Takao Fujii, Masahide Hamaguchi, Moritoshi Furu, Hiromu Ito, Chikashi Terao, Keiichi Yamamoto, Wataru Yamamoto, Takashi Matsuo, Masato Mori, Koichiro Ohmura, Hiroshi Kawabata, Tsuneyo Mimori

**Affiliations:** 1 Department of the Control for Rheumatic Diseases, Graduate School of Medicine, Kyoto University, Kyoto, Japan; 2 Department of Rheumatology and Clinical Immunology, Graduate School of Medicine, Kyoto University, Kyoto, Japan; 3 Department of Experimental Immunology, Immunology Frontier Research Center, Osaka University, Osaka, Japan; 4 Department of Orthopedic Surgery, Graduate School of Medicine, Kyoto University, Kyoto, Japan; 5 Center for Genomic Medicine, Graduate School of Medicine, Kyoto University, Kyoto, Japan; 6 Division of Clinical and Epidemiological Database, Department of Information Governance, National Cerebral and Cardiovascular Center, Osaka, Japan; 7 Department of Health Information Management, Kurashiki Sweet Hospital, Kurashiki, Japan; 8 Department of Hematology and Oncology, Graduate School of Medicine, Kyoto University, Kyoto, Japan; Institut national de la santé et de la recherche médicale (INSERM), France

## Abstract

**Objective:**

To compare the effect of tocilizumab (TCZ) with other biologic therapies in improving anemia of rheumatoid arthritis (RA) patients.

**Methods:**

We compared the change of hemoglobin (Hb) levels in a cohort of 147 consecutive RA patients who were treated with biologics for more than 12 weeks. Twenty eight patients were treated with TCZ, and 119 patients were treated with biologics other than TCZ (87 with TNF inhibitors and 32 with abatacept). The change of Hb levels from baseline to week 12 was compared between the TCZ and the non-TCZ groups. We performed univariate and multivariate analyses with adjustment of potential confounders such as baseline characteristics, concomitant treatment, and the clinical response to treatment.

**Results:**

Hb levels generally increased after biologic therapies both in the TCZ and the non-TCZ groups. The increase of Hb levels was greater in the TCZ group than in the non-TCZ groups (1.1 g/dL in the TCZ group vs 0.3 g/dL in the non-TCZ group, p = 0.009). Univariate analysis revealed that increase of Hb levels was also significantly associated with lower Hb, higher Low Hemoglobin Density, and higher CRP levels at baseline and greater reduction in the clinical disease activity index. TCZ therapy was significantly associated with the increase of Hb levels even after adjustment for these factors by multivariate analysis (p<0.001, effect size 0.08–0.12).

**Conclusion:**

TCZ therapy is an independent factor associated with the increase of Hb level after biologic therapies in RA patients. It will help in selecting appropriate biologics for RA patients with anemia.

## Introduction

Anemia is an important complication in rheumatoid arthritis (RA) which is associated with physical disability and increased mortality [1] [2]. Anemia of chronic disease (ACD), also known as anemia of chronic inflammation, is the major reason for developing anemia in RA patients. Recent studies suggest that ACD develops via multiple mechanisms including pathogenic iron homeostasis, impaired erythropoiesis, and the blunted erythropoietin response [1]. Inflammatory cytokines such as IL-6 and tumor necrosis factor α (TNFα) are critically involved in its process. For example, IL-6 induces hepcidin, a critical regulator of iron metabolism in ACD [3] [4], while TNFα and IL-1 impair erythropoiesis and induce the blunted erythropoietin response [5]. Among various mechanisms, recent studies suggest the central role of hepcidin and IL-6 for the pathophysiology of ACD [3].

Recently, the treatment of RA has significantly advanced over past decade by the introduction of biologic therapies (biologics) [6]. Today, several biologics that target different molecules are used in RA clinical practice with comparable efficacy and side effects [6]. These include TNF inhibitors (infliximab, etanercept, adalimumab, golimumab, and certolizumab); an IL-6 receptor antagonist, tocilizumab (TCZ); a T cell co-stimulatory blocker, abatacept; and a B cell specific depletor, rituximab, and so on. Because IL-6 and other cytokines are critically involved in the pathogenesis of ACD, biologic therapies such as TCZ and TNF inhibitors could potentially increase hemoglobin (Hb) levels after treatment. Indeed, increase of Hb levels after biologic therapies has been demonstrated in RA and in other inflammatory diseases [5] [7] [8] [9] [10] [11] [12].

Given the central role of IL-6 in ACD, it is possible that TCZ therapy would improve anemia more effectively than other biologics. However, it should be determined carefully because the etiology of anemia in RA is multifactorial and ACD is not the only cause of anemia in RA. The Hb levels after treatment may be influenced by various factors such as baseline characteristics, concomitant treatment, or clinical response to treatment. For example, age, sex, renal function, or initial levels of Hb and inflammatory markers would apparently affect the change in Hb levels [13] [14]. Methotrexate treatment frequently causes folate deficiency and macrocytic anemia, while the use of non-steroidal anti-inflammatory drugs (NSAIDs) or glucocorticoids might cause gastrointestinal bleeding that may lead to iron deficiency and microcytic anemia. The change in Hb levels may correlate with the clinical response to treatment or the reduction of inflammatory markers. Therefore, clinical research is necessary to determine which biologics are optimal in improving anemia of RA. Because anemia is associated with not only the patient's subjective symptoms such as fatigue [2], but also with the radiological progression of RA [15], such studies will be beneficial for clinicians aiming at remission of RA.

The aim of this study is to determine whether TCZ therapy is more effective at increasing Hb levels in RA than other non-TCZ biologics even after taking into account many confounding factors. For this aim, we conducted a cohort study by enrolling the consecutive RA patients treated by biologics in our cohort, and analyzed the effect of TCZ therapy with potential confounders for the increase of Hb levels.

## Patients and Methods

### Study design and population

The study population was enrolled from Kyoto University Rheumatoid Arthritis Management Alliance (KURAMA) cohort and followed up prospectively [16]. Briefly, the KURAMA cohort was established on 1^st^ May 2011 in the Center for Rheumatic Diseases in Kyoto University Hospital aiming at the tight control of RA following recent advances in RA treatment and to utilize their sequential clinical and laboratory data for clinical investigations [6] [17]. Annually, around 400 patients are consecutively registered in the KURAMA cohort. The median age of the patients is 64.5 years old, 87.6% female, median disease duration is 13.0 years [17]. In this cohort, around 30% of the patients are on the treatment with biologics which are initiated not only for patients with high disease activity but also for patients with low to moderate disease activity to suppress the residual joint swelling [17].

In the current study, we enrolled all the patients consecutively from 1^st^ May 2011 to 1^st^ November 2012. Inclusion criteria were as follows: patients with written informed consent, over 18 years of age, fulfilling the American College of Rheumatology / European League Against Rheumatism classification criteria for RA in 2010 [18], and newly introduced for biologics in our institute during the study period and remained on the drug for more than 12 weeks. Patients receiving hemodialysis or erythropoietin therapy and patients who had an active gastrointestinal bleeding event during the study period were excluded. The study was designed in accordance with the declaration of Helsinki and was approved by the ethics committee of Kyoto University Graduate School and Faculty of Medicine. We followed up all the included patients for 12 weeks, and analyzed the change of Hb levels and disease conditions. Biologics used in this study were TCZ, infliximab, etanercept, adalimumab, golimumab, and abatacept. Rituximab and certollizumab were not included because they were not approved for RA patients in Japan at the time of this study.

### Data collection

Clinical data included age, sex, body-mass index, disease duration of RA, swollen joint count, tender joint count, patient's global assessment of disease activity, and physician's global assessment of disease activity. Serological data and blood cell count included Hb, red blood cell count (RBC), mean corpuscular volume (MCV), mean corpuscular hemoglobin (MCH), mean corpuscular hemoglobin concentration (MCHC), and C-reactive protein (CRP). Anemia was defined as Hb level <12.0 in women and <13.0 in men. Iron status in the presence of inflammation was assessed by Low Hemoglobin Density (LHD%) transformed from MCHC, because LHD%>5.5 is a reliable marker for detecting the functional iron deficiency in ACD patients [19] [20]. Renal function was assessed by estimated glomerular filtration rate calculated by creatinine value, sex and age. RA disease activity was evaluated by clinical disease activity index (CDAI) and CRP separately, because IL-6 inhibition has a prominent effect on the production of hepatic acute phase proteins such as CRP and it is preferred to use CDAI which does not comprise CRP for the assessment of the clinical response to TCZ [21]. Physical function was assessed by modified health assessment questionnaire-disability index (mHAQ). Clinical response to biologic therapies was assessed by the reduction of CDAI, CRP or mHAQ from baseline to week 12 (ΔCDAI  =  CDAI at baseline - CDAI at week 12, ΔCRP  =  CRP at baseline - CRP at week 12, ΔmHAQ  =  mHAQ at baseline – mHAQ at week 12). Data for the concomitant use of methotrexate, glucocorticoids, NSAIDs, iron drugs, and folic acid were also recorded. Clinical and laboratory data were compared at baseline and week 12 after the initiation of biologic therapies.

### Statistical analysis

The SPSS statistical package, version 11.0.1 J (SPSS, Inc., Chicago, IL) was used for all the statistical analyses and P value less than 0.05 was considered statistically significant. Continuous variables in two groups were assessed by Mann-Whitney u-test and frequencies were assessed by Chi square test. Univariate regression analysis was performed to analyze associations between the increase of Hb and potential confounders. The relationship between the increase of Hb and potential confounders were evaluated using Spearman's Rank correlation coefficient. Multivariate analysis of covariance was used to analyze the effect size TCZ therapy and other confounding factors displaying significant associations in the univariate analyses for the increase of Hb levels. Because the effect of biologic therapies on the increase of Hb levels was unknown, a formal sample size estimate was not made a priori. Data were expressed as median (min to max) for continuous variables and percentages (numbers) for categorical variables unless otherwise mentioned.

## Results

### Study population

During the study period, 750 patients were registered in the KURAMA cohort and 151 patients were newly introduced for biologic therapies and remained on the drugs for more than 12 weeks. Three patients receiving hemodialysis and one patient who had gastrointestinal bleeding complication before week 12 were excluded from the study. Among the 147 study population, 28 (19.0%) patients were treated with TCZ and 119 (81.0%) patients with non-TCZ biologic therapies; 87 (59.2%) patients were treated with TNF inhibitors including 23 (15.6%) patients with etanercept, 27 (18.4%) patients with infliximab, 9 (6.1%) patients with adalimumab, 28 (19.0%) patients with golimumab, while 32 (21.8%) patients were treated by abatacept. The study population (n = 147) were divided into the TCZ group (n = 28) and the non-TCZ group (n = 119) including TNF inhibitors(infliximab, etanercept, adalimumab, golimumab, n = 87) and abatacept (n = 32) and used for the analysis.

### Baseline characteristics and clinical response

The baseline characteristics including Hb, MCV, MCH, LHD%, or CRP levels and CDAI were not different between the TCZ and the non-TCZ groups (**Table 1**). Anemia was present in 53.6% of the TCZ group and 47.9% of the non-TCZ group. The median levels of MCV in anemic patients were normal (91.3), but the minimum and maximum levels of MCV varied widely (69.6–105.1) suggesting that the causes of anemia in this study population are multifactorial. LHD% levels exceeded the cut off value 5.5 in 81.0% of the patients suggesting the presence of functional iron deficiency in the majority of patients. Baseline mHAQ tended to be higher in the TCZ group than in the non-TCZ group, but this tendency was not found to be statistically significant. The frequency of concomitant use of methotrexate or NSAIDs was not different between the two groups, while that of the use of glucocorticoids and iron drugs was higher in the TCZ group than in the non-TCZ group. Additionally, previous use of biologics was more frequent in the TCZ group than in the non-TCZ group (**Table 1**). The clinical responses to treatment assessed by ΔCDAI, ΔCRP, and ΔmHAQ were not different between the TCZ and the non-TCZ group (**Table 1**).

**Table 1:** Characteristics of the study population and increase of Hb levels after biologic therapies.

	TCZ group	non-TCZ group	P
	N = 28	N = 119	
**Basic characteristics**			
Age, year	62 (34–)	60 (19–86)	0.58
Female, % (n)	78.6% (22)	88.2% (105)	0.22
Duration of RA, month	127 (9–464)	77 (3–759)	0.22
BMI, kg/m^2^	23.0 (16.6–32.5)	22.2 (0.2–32.5)	0.48
eGFR	78.6 (35.9–122.4)	80.7 (28.8–153.4)	0.50
Hb, g/dl	12.3 (6.3–15.9)	12.1 (8.3–16.7)	0.99
RBC, 10^6^/ml	4.0 (2.6–5.3)	4.1 (2.7–5.4)	0.32
MCV	94.0 (77.2–104.9)	92.3 (69.6–105.1)	0.44
MCH, pg	30.4 (23.2–35.6)	29.9 (20.0–35.6)	0.34
LHD%	12.0 (1.5–82.0)	11.5 (1.2–94.7)	0.89
LHD%>5.5, % (n)	78.6% (22)	81.5% (97)	0.79
CDAI	17.4 (0.9–62.4)	12.2 (1.2–49.6)	0.12
CRP, mg/dl	0.4 (0–11.9)	0.5 (0–9.2)	0.67
mHAQ	0.8 (0–2.5)	0.4 (0–2.6)	0.052
Anti-CCP positivity, % (n)	91.7% (22)	91.3% (73)	1.00
**Concomitant treatments**			
Methotrexate, % (n)	64.3% (18)	73.9% (88)	0.35
Glucocorticoids, % (n)	75.0% (21)	42.9% (51)	0.003
NSAIDs, % (n)	71.4% (20)	54.6% (65)	0.14
Iron drugs, % (n)	10.7% (3)	5.9% (7)	0.40
Folic acid, % (n)	57.1% (16)	70.6% (84)	0.18
Previous biologics, % (n)	71.4% (20)	31.1% (37)	<0.001
**Clinical response**			
ΔCDAI	7.2 (−2.2–44.3)	6.2 (−10.0–33.5)	0.52
ΔCRP (mg/dl)	0.2 (−0.6–10.2)	0.2 (−6.7–6.7)	0.24
ΔmHAQ	0.125 (−1–1.3)	0.125 (−2.6–1.5)	0.33
**Increase of Hb levels, g/dl**			
	1.1 (−1.2–4.7)	0.3 (−1.7–2.4)	0.009

Continuous variables were compared by Mann-Whitney u-test and were expressed as median (min-max). Categorical variables were compared by Chi square test and were expressed as % (number) unless otherwise mentioned. Clinical response to biologic therapies was assessed by the reduction in CDAI, CRP or mHAQ from baseline to week 12. Increase of Hb levels (g/dl) was compared between the TCZ and the non-TCZ group and was expressed as median (Min-Max). TCZ, tocilizumab; RA, rheumatoid arthritis; BMI, body mass index; eGFR estimated glomerular filtration rate; Hb, hemoglobin; RBC, red blood cell count; MCV, mean corpuscular volume; MCH, mean corpuscular hemoglobin; LHD%, low hemoglobin density; CDAI, clinical disease activity index; CRP, C-reactive protein; mHAQ modified health assessment questionnaire-disability index; CCP, citrullinated protein/peptide; NSAIDs, non-steroidal anti-inflammatory drugs.

### Increase of Hb levels after TCZ therapy

In the current study, we aimed to compare the effects of biologic therapies on anemia of RA. The increase of Hb levels was greater in the TCZ group than in the non-TCZ group (1.1 vs 0.3 g/dL, respectively, p = 0.009) (**Table 1**), while no significant difference was observed within the non-TCZ group between TNF inhibitors (infliximab, etanercept, adalimumab, golimumab) and abatacept (0.4 vs 0.2 g/dL, respectively, p = 0.64). 

**Figure 1:**
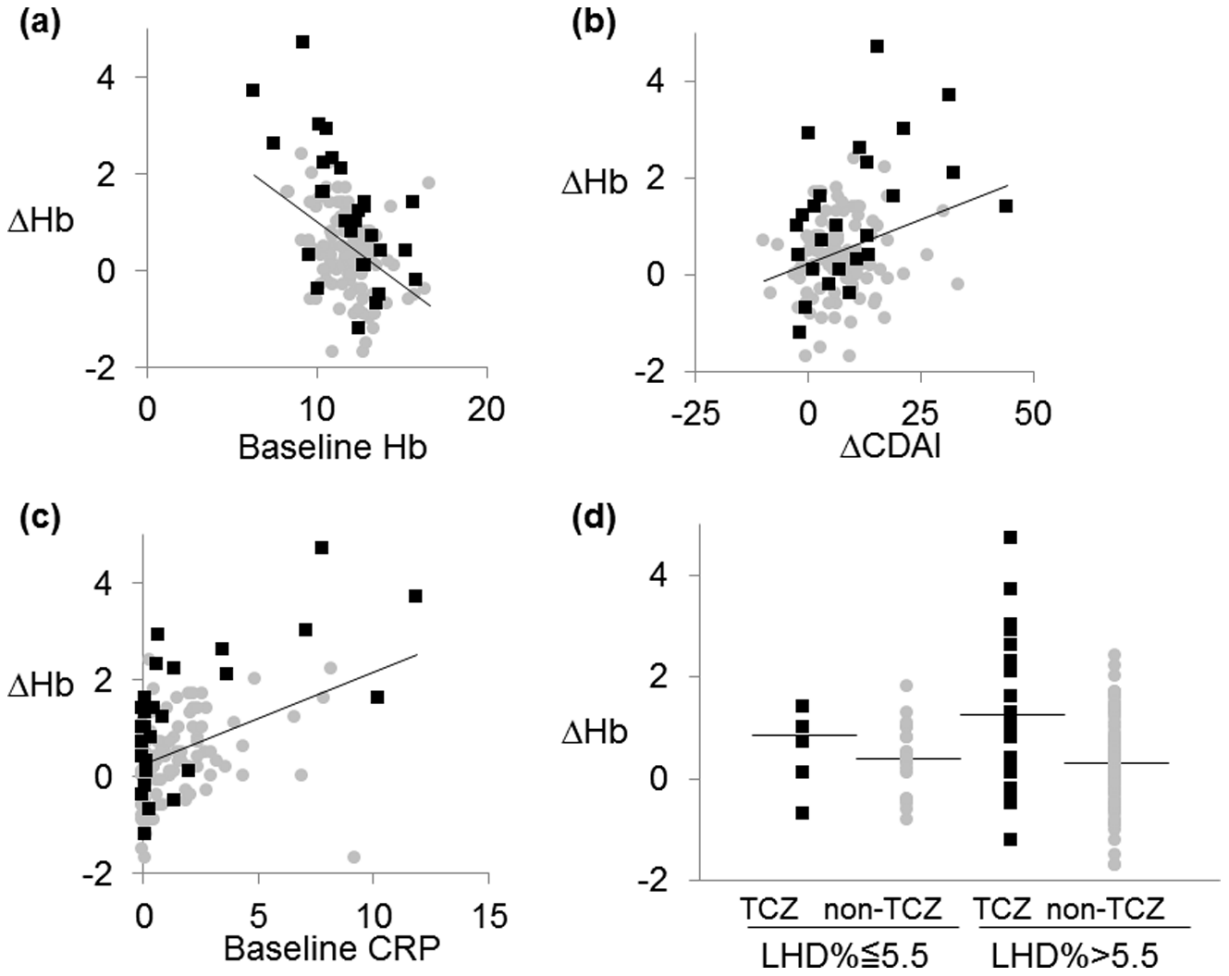
Correlation between the increase of hemoglobin levels and baseline hemoglobin (a), CRP (b), reduction in the clinical disease activity index (c), and baseline LHD% (d). Dot plot suggests the correlation between (a) baseline hemoglobin (Hb) and ΔHb (N = 147), (b) baseline CRP and ΔHb (N = 147), and (c) reduction in the clinical disease activity index (ΔCDAI) and ΔHb (N = 122) in rheumatoid arthritis patients treated by TCZ (black closed square) or non-TCZ biologics (gray closed circle). The straight line corresponds to the best-fit linear regression line for all samples. (d) ΔHb levels in patients treated with TCZ (black closed square) or non-TCZ biologics (gray closed circle) are expressed in two groups divided by the baseline LHD% value 5.5. The straight lines represent the median levels of ΔHb for indicated groups. (ΔHb  =  Hb at week 12 - Hb at baseline, ΔCDAI  =  CDAI at baseline – CDAI at week 12; LHD% =  low hemoglobin density.)

Next, we performed univariate regression analysis to determine if there were any confounding factors associated with the increase of Hb levels after biologic therapies. Increase in Hb levels was significantly correlated with lower Hb levels, higher LHD%, and higher CRP levels at baseline, and with better clinical response assessed by ΔCDAI or ΔCRP, but not ΔmHAQ (**Table 2**) (**Figure 1**). Increase of Hb levels was not associated with other continuous variables such as age, body mass index, MCV, or renal function assessed by estimated glomerular filtration index. Furthermore, categorical variables such as concomitant use of methotrexate, glucocorticoids, NSAIDs, iron drugs, folic acid, or previous use of biologics did not affect the increase in Hb levels (**Table 2**).

**Table 2:** Factors associated with increase of Hb levels after biologic therapies.

	ρ	P
**Basic characteristics**		
Age, year	0.00	0.97
Sex (female)	−0.06	0.49
Duration of RA, month	−0.06	0.54
Body mass index, kg/m^2^	0.05	0.58
Estimated glomerular filtration index	0.10	0.29
Hb, g/dl	−0.37	<0.001
RBC, 10^6^/ml	−0.34	<0.001
MCV, fL	−0.02	0.80
MCH, pg	−0.10	0.29
LHD%	0.21	0.01
CDAI	0.03	0.77
CRP, mg/dl	0.32	<0.001
mHAQ	−0.07	0.51
**Concomitant treatments**		
Methotrexate	0.02	0.79
Glucocorticoids	0.04	0.68
NSAIDs	−0.14	0.09
Iron drugs	0.10	0.25
Folic acid	0.09	0.27
Previous biologics	−0.14	0.10
**Clinical response**		
ΔCDAI	0.23	0.01
ΔCRP, mg/dl	0.34	<0.001
ΔmHAQ	0.18	0.09

Association between the increase of Hb levels and characteristics of the patients were assessed by Spearman's rank test. Clinical response to biologic therapies was assessed by the reduction of CDAI, CRP, or mHAQ from baseline to week 12, (ΔCDAI  =  CDAI at baseline – CDAI at week 12, ΔCRP  =  CRP at baseline – CRP at week 12, and ΔmHAQ  =  mHAQ at baseline – mHAQ at week 12, respectively). ρ represents Spearman's rank correlation coefficient; RA, rheumatoid arthritis; BMI, body mass index; eGFR, estimated glomerular filtration index; Hb; hemoglobin, RBC; red blood cell count; MCV, mean corpuscular volume; MCH, mean corpuscular hemoglobin; LHD%, low hemoglobin density, CDAI, clinical disease activity index; CRP, C-reactive protein; mHAQ, modified health assessment questionnaire-disability index; NSAIDs, non-steroidal anti-inflammatory drugs.

Thus, we selected baseline Hb levels, baseline LHD%, baseline disease activity, and clinical response to treatment as potential confounders for the multivariate analysis (**Table 3**). Because LHD%>5.5 was a reliable marker for detecting functional iron deficiency, the dichotomous variable with a cut off value LHD%>5.5 was used in the multivariate analysis. Baseline disease activity and clinical response was assessed by CDAI and ΔCDAI in model 1, CRP and ΔCRP in model 2, respectively. In both models, TCZ remained as an independent factor associated with the increase of Hb level even after adjustment for baseline Hb levels, baseline LHD%>5.5, baseline disease activity and the clinical response to treatment. The effect size of TCZ for the increase of Hb level was 0.08 to 0.12 (**Table 3**). Baseline Hb levels, baseline LHD%>5.5, disease activity and clinical response to treatment also remained as a significant factor for the increase of Hb, suggesting that not only TCZ use but also baseline anemic state, functional iron deficiency, and the state of inflammation collectively determine the change of Hb levels after biologic therapies (**Table 3**).

**Table 3:** Effect of TCZ therapy and other confounding factors for the increase of Hb levels.

	Model 1		Model 2	
	Effect size	P	Effect size	P
**TCZ therapy**	0.12	<0.001	0.08	<0.001
**Hb level at baseline**	0.23	<0.001	0.13	0.001
**LHD%>5.5 at baseline**	0.06	0.009	0.03	0.028
**Disease activity at baseline**	0.08	0.002	0.01	0.40
**Clinical response**	0.12	<0.001	0.08	<0.001

The effect size of TCZ therapy and other confounding factors for the increase of Hb levels after treatment was analyzed by the multivariate analysis of covariance. Disease activity and clinical response was assessed by CDAI and ΔCDAI in model 1, and CRP and ΔCRP in model 2, respectively. (ΔCDAI  =  CDAI at baseline – CDAI at week 12, ΔCRP  =  CRP at baseline – CRP at week 12). TCZ, tocilizumab; Hb, hemoglobin; LHD%, low hemoglobin density; CDAI, clinical disease activity index; CRP, C-reactive protein; mHAQ, modified health assessment questionnaire-disability index.

To exclude the possible impact of iron supplementation in our cohort, we also analyzed the data by excluding the patients who received iron drugs from the study population (excluding 3 patients in TCZ group and 7 patients in the non-TCZ group, N = 137). The increase of Hb levels was also significantly higher in the TCZ group than in the non-TCZ group (1.2 vs 0.3 g/dl, respectively, p = 0.0003), and the multivariate analysis revealed that TCZ therapy was an independent factor associated with the increase of Hb levels (effect size 0.12–0.15).

## Discussion

In this study, we have shown that Hb levels increase more after TCZ therapy than other biologic therapies. Even after adjusting for baseline characteristics, baseline disease activity, and clinical response to treatment, TCZ therapy was an independent factor that was associated with the increase of Hb levels after biologic therapies. Improvement of anemia after biologic therapies such as TCZ or TNF inhibitors has been reported previously in RA [5] [8] [11], and other inflammatory diseases such as Castleman's disease [9] [10]. However, few studies have compared the effect of different biologic therapies with possible confounding factors for the increase of Hb levels. We have recently reported the pronounced increase of Hb levels by TCZ than TNF inhibitors in RA patients in a different cohort [22]. However, in that cohort, baseline CRP levels and clinical responses were higher in the TCZ group than the TNF inhibitor's group and the effect of these confounding factors were not fully investigated [22]. Our study suggests that TCZ therapy would be beneficial for treating anemia of RA patients, in particular with severe anemia and high disease activity.

Our results are consistent with the recent studies suggesting the central role of IL-6-hepcidin axis in ACD [3] [23]. Hepcidin, a peptide hormone produced mainly by hepatocytes, reduces intestinal iron absorption and blocks iron release from macrophages to induce hypoferremia and pathogenic iron homeostasis to cause ACD [3] [4] [24]. IL-6 was the necessary and sufficient cytokine for the induction of hepcidin during inflammation because IL-6 or hepcidin knockout mice do not develop anemia upon inflammation [23] [25]. IL-6 level was the only factor other than disease stage that independently predicted Hb levels in cancer associated ACD [26]. IL-6 inhibition by TCZ was effective in correcting anemia in Castleman's disease which is characterized by the overproduction of IL-6 from lymph nodes [9]. Therefore, IL-6-hepcidin axis is central to ACD both *in vivo* and *in vitro*.

Although we have shown that TCZ therapy was an independent factor associated with the increase of Hb levels after biologic therapies by consecutively enrolling all the RA patients treated by biologics in the KURAMA cohort, our study has several limitations. First, since the study population was small, the result may not be valid for all RA patients. Second, several baseline characteristics such as concomitant use of glucocorticoids and iron drugs were different between the TCZ and the non-TCZ group in our study population, although these factors did not affect the increase of Hb levels in the univariate analysis and the result of the multivariate analysis was not affected when these factors were included as covariates. Third, due to the lack of data on markers of iron status (serum iron, ferritin, and transferrin), hepcidin, or erythropoietin, we could not assess the mechanisms of anemia in our study population. Although we postulate that ACD is the major mechanism involved because IL-6 inhibition by TCZ was effective in increasing Hb levels, we cannot rule out other possibilities; for example, IL-6 inhibition may have acted directly on erythropoiesis or the erythropoietin response [27]. Further studies will be necessary to fully determine the role of IL-6 in anemia of RA.

In conclusion, this study has shown that Hb level significantly increases after TCZ therapy compared with non-TCZ biologic therapies even after adjustment for baseline characteristics, baseline disease activity, and clinical response to treatment. It implies the central role of IL-6 in anemia of RA patients and suggests that TCZ would be a good option for RA patients with anemia.
